# The Comparison of Double J Stent Insertion and Conservative Treatment Alone in Severe Pure Gestational Hydronephrosis: A Case Controlled Clinical Study

**DOI:** 10.1155/2014/989173

**Published:** 2014-01-20

**Authors:** Kürşat Çeçen, Kahraman Ülker

**Affiliations:** ^1^Departments of Urology, Kafkas University School of Medicine, 36000 Kars, Turkey; ^2^Obstetrics & Gynecology, Kafkas University School of Medicine, 36000 Kars, Turkey

## Abstract

*Objective*. Management options of gestational hydronephrosis are based on the coexisting stone disease, pyelonephritis, and renal disease. However, the management option and its consequences in the absence of a coexisting disease state are not clear. In this study we aimed to compare the effectiveness of conservative treatment and double J insertion in symptomatic pure gestational hydronephrosis. *Material and Methods*. The data of the women with severe pure gestational hydronephrosis over a nine-year period was collected retrospectively. The included women were grouped into two according to receiving double J stent insertion or conservative treatments. *Results*. Double J insertion and conservative treatment groups included 24 and 29 women, respectively. Hydronephrosis was demonstrated on the right, left, or both kidneys in 37 (70%), 13 (24%), and 3 (6%) women, respectively. None of the participants gave birth prior to the 37th week. The demographics, initial pain scores, the severity of the hydronephrosis during first admission, and pain scores one week after the interventions did not differ significantly between groups (*P* > 0.05). Similarly, the rates of complications, postpartum pain scores, and permanent hydronephrosis did not differ between groups (*P* > 0.05). *Conclusion*. Double J insertion in symptomatic pure gestational hydronephrosis adds no benefit to conservative treatment.

## 1. Introduction

Dilatation effect of the progesterone and mechanical compression of the enlarging uterus result in hydronephrosis of the pregnancy. In most of the pregnancies hydronephrosis is considered as a “normal” finding of the pregnancy. It is more frequently observed on the right side and can be demonstrated by ultrasound beginning from the second trimester and it may be present until the 12th postpartum week [1].

Although hydronephrosis of pregnancy can be present in up to 80% of the pregnancies [2] the management options are not clearly defined. The authors of the previous studies suggested conservatory treatments unless the symptoms persist and additional complications including resistant urinary tract infection and deterioration of the renal function had occurred [3–5].

Most of the management options of gestational hydronephrosis are based on the coexisting stone disease, pyelonephritis, and renal disease. However, the management option and its consequences in the absence of a coexisting disease state are not clear. In a previous study conducted after the randomization of the moderate-severe hydronephrosis, the insertion of double J stent was found more effective than conservative therapy alone [6]. However, interestingly the authors suggested the conservative treatment as the first choice depending on the complications and discomfort related to the surgical treatment. In addition, in most of the studies neither the degree of hydronephrosis nor the severity of the discomfort was thoroughly assessed.

In this retrospective study we aimed to compare the effectiveness of conservative treatment and double J insertion in symptomatic “pure gestational hydronephrosis”.

## 2. Material and Methods

Following the approval of local ethical committee of Kafkas University School of Medicine the records of patients diagnosed with hydronephrosis were evaluated. The study included pregnant women diagnosed with hydronephrosis within the departments of obstetrics and gynecology and referred to the department of urology between 2004 and 2013 in Kars State Hospital, Kars SSK Hospital, and Kafkas University School of Medicine.

The study included singleton pregnancies without identifiable maternal and fetal complications other than maternal hydronephrosis and severe flank pain. Active urinary tract infection, urolithiasis, known genitourinary anomalies, gestational or nongestational diabetes mellitus, preeclampsia or chronic hypertension, chronic kidney or liver diseases, and chronic vascular or connective tissue diseases caused exclusion. The well-being of the fetus and the pregnancy was established with a biophysical profile scoring. Pregnancies with a jeopardized fetus, Bishop score over four, or active labor were excluded. Gestational age was established by the first date of the last menstrual period and confirmed by the findings of first trimester ultrasound examinations.

The demographic data including the maternal age, gravidity, parity and abortion numbers, and the outcome of the previous pregnancies were obtained at the time of first admission. In order to determine the severity of the perceived pain we used the 11-point (0, 1, 2, 3, 4, 5, 6, 7, 8, 9, 10; 0 and 10 points as no pain and maximum pain, resp.) numerical rating scale (NRS).

Following the initial evaluation, all patients had urine analysis, complete blood count, and serological and biochemical tests including the liver and kidney function tests. Our study included the pregnancies without remarkable laboratory results during first admission. Ultrasound examinations were repeated to determine the severity of the hydronephrosis and we included only the severe hydronephrosis defined by Zwergel et al. [4]. Magnetic resonance imaging (MRI) examination was not an option until the last four years; thus we excluded the results of the MRI examination during the evaluation of the data.

We recommended the insertion of a double J stent to all pregnant patients with a severe hydronephrosis and a NRS pain score of ≥5 and provided information about the complications and side effects of the insertion of a double J stent. Some of the patients accepted the insertion of the double J stent; however some refused it. Double J insertion was performed under general anesthesia and all participants were hospitalized at least one day following the intervention. The stents were scheduled to be removed at the 2nd postpartum month.

Double J stent was inserted or not; all patients had a repeat urological visit one week later and the visits were repeated every 15 days in patients with unremarkable additional findings. All the evaluation tests were repeated in the proceeding visits. In case where additional complications or complaints detected the patients were treated appropriately. The study included the analysis of the severity of pain scores one week after the initial admission and at the sixth postpartum week. The severity of hydronephrosis was also evaluated at the sixth postpartum week by ultrasound examination. The women with a calyceal diameter of >5 mm during the postpartum period were reevaluated by the appropriate use of computed tomography, intravenous pyelography, voiding cystography, or retrograde pyelography in order to exclude urinary tract anomalies and stones.

All patients in the double J stent or conservative treatment groups were instructed to rest as possible as they could on the opposite site of where the hydronephrosis was demonstrated and drink at least two liters of water per day. Two doses of 75 mg diclofenac sodium I.M. with 12-hour intervals were used during the initial management of pain and oral metamizole and/or paracetamol were used during the following days.

Patients with complications like urinary tract infection, urine culture positiveness, or pyelonephritis detected in urine analysis or urine culture were treated appropriately. Kidney function deterioration was defined with the elevation of blood urea nitrogen and creatinine levels over the defined laboratory levels. All patients with the findings of kidney function deterioration and pyelonephritis were hospitalized, followed, and treated appropriately.

During the evaluation phase 53 pregnant women diagnosed with pure hydronephrosis were selected for statistical analysis. SPSS version 16.0 software (SPSS Inc, Chicago, IL) was used to interpret the data and perform the statistical tests. During the comparison of the data obtained from the two groups we used Student *t* test if the distribution was normal and Mann Whitney *U* test if the distribution was nonnormal. During the comparison of the NRS pain scores during the initial admission, one week after the initial admission and at the sixth month visit we used repeated measures analysis of variance and paired *t* tests. The relation among the variables was studied with Pearson's correlation test. A *P* value <0.05 was considered significant.

## 3. Results

A total of 53 pregnant women diagnosed with hydronephrosis were included in the study. Although we had offered all participants the insertion of double J stents, of the 53 participants only 24 (45%) accepted the interventions and 29 (55%) of them insisted on conservative treatment. We did not observe any serious complication during or after the insertion of double J stent. All the stents were removed at the scheduled time. None of the patients in the conservative group required a stent after the initial conservative treatment.

Hydronephrosis was demonstrated on the right, left, or both kidneys in 37 (70%), 13 (24%), and 3 (6%) women. All bilateral hydronephrosis cases were in the conservative group. Double J stent was inserted into the left and right sides in seven (7/13, 54%) and 17 (17/37, 46%) women, respectively.

None of the participants gave birth prior to 37th week of their gestation. During the postnatal second month visit, it was confirmed that all participated women had healthy infants without remarkable problems. Severe hydronephrosis (>15 mm dilatation) at the second postpartum month was demonstrated in three of the participants (one in double J group and two in conservative group). The postpartum maternal hydronephrosis was demonstrated on the right side in the woman with double J stent. One right sided and one left sided postpartum hydronephrosis were observed in the conservative group.

Patients with postpartum hydronephrosis were scheduled on a follow-up program and followed appropriately. The women with a calyceal dilatation over 5 mm were reevaluated by using additional diagnostic tests.

The data dealing with the demographics, initial NRS scores, and the severity of the hydronephrosis at the time of first admission was summarized in Table 1. The mean admission time of the symptomatic hydronephrosis of the pregnancy was around the 24th week in both groups. The demographic data including the maternal age, gestational week, gravidity, parity, and abortion numbers did not differ between the groups followed conservatively or after the insertion of double J stent (*P* < 0.05). Similarly, hydronephrosis rate during previous pregnancy and the maximal calyceal diameter and NRS pain scores during admission were not significantly different in both groups (*P* < 0.05).

All participants were reevaluated one week later. Repeated measures of analysis of variance and paired *t* tests suggested that the initial NRS pain scores were significantly decreased following the conservative treatments and double J stent insertion (*P* < 0.05). Moreover, NRS pain scores at the postpartum sixth week were significantly lower than the NRS pain scores one week after the first admission (*P* < 0.05). However, similar to the pain scores during first admission, the NRS pain scores of conservative treatment and double J stent groups (Table 2) were not significantly different at either one week after the first admission or the sixth postpartum week (*P* > 0.05).

During the follow-up period of the pregnant women with gestational hydronephrosis, the first week following the admission was uneventful. However, although the number and percentage of the complications did not differ significantly between the study groups (*P* < 0.05), following the first week some women had urinary tract infection, urine culture positiveness, pyelonephritis, and mild deterioration of the kidney functions (Table 2). Appropriate treatment with antibiotics, positioning, and hydration after hospitalization overcame the complications satisfactorily.

During correlation analysis it was observed that the maternal age, gravidity, and parity, and abortion numbers correlated with each other (*P* < 0.05). The severity of the maternal hydronephrosis (>15 mm) did not correlate with the gravidity, parity and abortion numbers, the gestational week, the presence of maternal hydronephrosis in the previous pregnancy, and the initial NRS pain scores (*P* > 0.05). The continuity of hydronephrosis at the postpartum period was significantly correlated with the gravidity, parity and abortion numbers, pyelonephritis, presence of hydronephrosis in the previous pregnancy, and the NRS pain scores during the postpartum period (*P* < 0.05).

## 4. Discussion

### 4.1. Principle Findings

The management of severe pure gestational hydronephrosis by conservative treatment or insertion of double J stent had similar results. It seemed that insertion of a double J stent added no benefit over conservative treatment. The symptoms of severe pure gestational hydronephrosis improved after conservative or double J insertion treatments. Pure gestational hydronephrosis did not worsen the outcome of the pregnancies.

### 4.2. Strengths of the Study

To our knowledge this is the first study comparing the effect of the insertion of the double J stent with the conservative treatment in pure gestational hydronephrosis. We included the same patient groups and the only changed variable was the presence or absence of double J stent. In addition, although the pain sensation is unavoidably subjective to a degree, we used NRS pain scores and using NRS pain scores increases the objectiveness of the pain assessment. Moreover, most of the previous studies did not comprehensively define the strict criteria in which the double J insertion was indicated. Generally it was accepted that in cases where the conservative measures such as intravenous hydration, analgesics, and antibiotic therapy were unsuccessful to overcome the symptoms, particularly sepsis or compromised renal functions, ureteral stent placement might be needed [7]. We offered the insertion of double J stent to all symptomatic gestational hydronephrosis patients with a largest calyceal diameter of ≥15 mm and NRS pain scores of ≥5. Thus, the objectiveness of the indication for the insertion of double J stent was increased.

### 4.3. Limitations

Although we had a comparative control group, our study was retrospective. We included the severe pure gestational hydronephrosis; however the term “pure hydronephrosis” could be ascertained only after the postpartum examinations revealing the absence of other obstructive pathologies. On the one hand, pregnancy limits the use of some diagnostic tests resulting in an unintended radiation exposure of the fetus and some interventional tests which increases the risk of preterm labor and pregnancy related complications. On the other hand, the use of risky tests does not always ascertain the final and accurate diagnosis. Thus, in a prospective manner it is nearly impossible and impractical to diagnose the pure hydronephrosis with a 100% certainty.

Although we used the predefined calyceal diameter values [4] to classify the severity of the gestational hydronephrosis, there is a wide variability [8–11] of hydronephrosis definition which causes the reported occurrence varying between 43% and 100% [12]. In the study conducted by Faundes et al. a normal curve of dilatation of the urinary tract was proposed and the upper limits of maternal calyceal diameters were defined over 15 mm at some gestational weeks. To overcome the variability of the definition of hydronephrosis we included the symptomatic patients with a NRS pain score of ≥5. However, the reader should note that the use of a NRS pain scoring system also may cause subjectivity to a degree. Fortunately, the subjectivity of NRS may affect both the study and the control group.

The study included the symptomatic pregnant women with a largest calyceal diameter of ≥15 mm and a NRS pain score of ≥5, thus providing evidence for this population. However, the symptomatic patients with a largest calyceal diameter of <15 mm and a NRS pain score of ≥5, a largest calyceal diameter of ≥15 mm and a NRS pain score of <5, and a largest calyceal diameter of <15 mm and a NRS pain score of <5 were excluded. Thus the study results are unable to document the results of comparison of the effect of insertion of double J stent with conservative treatment in all symptomatic pure gestational hydronephrosis.

Although we performed the appropriate statistical tests to compare the study groups, we included a total of 53 patients. Thus, prospective studies including larger sample sizes are needed.

### 4.4. Comparison with the Previous Studies

Most of the publications related to gestational hydronephrosis included case reports or hydronephrosis associated with an additional obstructive disease [9, 13–17]. In all these publications the insertion of double J stent was found beneficial in terms of relieving the symptoms and treating the compromised renal function; however some of the publications were case reports and some were studies with additional obstructive diseases. In contrary our study included only the symptomatic patients with pure gestational hydronephrosis and we were unable to demonstrate any beneficial effect of inserting a double J stent. In addition, the mild deterioration of renal functions in the conservative treatment group was managed by hospitalization, close follow-up, positioning, intravenous hydration, and analgesics.

Rapid encrustation of the inserted stents due to the increased urinary calcium excretion during pregnancy was previously demonstrated [7]; however in our study all stents worked without any problem. Similar to our study results Sadan et al. [18] reported the use of stents during pregnancy without any significant problem. The mean gestational age on insertion of the stents was 29 weeks in their study but 24 weeks in our study. In addition, the stents remained in situ for 14 and 20 weeks in Sadan's and our studies, respectively. Similarly, Vendola et al. reported three pregnancy cases complicated with hydronephrosis continuing to term after the insertion of double J stent [19].

Although we did not experience any serious complication related to the insertion of double J stent during pregnancy, this does not mean that the procedure is completely safe and effective. Ringel et al. had to remove the 32% of the stents prior to the scheduled removal time because of side effects [20]. Frequency, urgency, dysuria, flank, or suprapubic pain is experienced by most of the patients with ureteral stents [21]. Urinary tract infection, migration of the stents, forgetting of the stent, and obstruction of the stent necessitating the removal or exchange of the device are additional possible complications and unwanted side effects [22–24].

In a previous study conducted by Tsai et al. [6] double J stent insertion (*n* = 25) was compared with the conservative treatment (*n* = 25) in gestational hydronephrosis. During the follow-up period five patients in the conservative treatment group did not effectively respond and the symptoms could only be relieved after the insertion of a double J stent. However, four patients complained of flank pain and stent discomfort. Thus, the insertion of double J stent caused the relief of symptoms in one extra patient. In our study none of the conservative treatment group patients had to receive surgical treatment during follow-up period. Even the deterioration of renal functions was relieved with hospitalization, intravenous hydration, and appropriate antibiotic use.

Although European Association of Urology recommends the use of the combination of nonsteroidal anti-inflammatory drugs (NSAID) and dipyrone as the first line treatment option during severe renal colic pain [25], continuous and prolonged use of NSAIDs may decrease fetal urine production and cause oligohydramnios. Thus, we preferred metamizole and paracetamol for pain relief and along with the other conservative measurements the pain scores were significantly relieved.

Depending on the findings of our study we recommend the conservative treatment as the first line option in pure gestational hydronephrosis. However, in order to ascertain the diagnosis, close follow-up with repeated diagnostic tests including urine analysis, urine culture, renal function tests, ultrasound, and MRI (where available) is mandatory.

## 5. Conclusions

Double J insertion in symptomatic pure gestational hydronephrosis seems to add no benefit over conservative treatment; thus it should be reserved for cases with complications or additional obstructive diseases.

## Figures and Tables

**Table 1:** The demographics, initial NRS pain scores, and the diameter of the largest pelvic calyces of the pregnancies complicated with hydronephrosis at the time of first admission.

	Double J inserted (*n* = 24)	Conservative treatment (*n* = 29)	*P* value
Gestational week at first admission	23.46 ± 3.22	24.72 ± 3.74	0.195*
Maternal age, years	24.12 ± 3.23	24.07 ± 3.78	0.955*
Gravidity	1	1	0.903**
Parity	0	0	0.753*
Abortion	0	0	0.903**
Hydronephrosis during previous pregnancy, *n* (%)	4 (17)	5 (17)	0.957*
The largest renal calyceal diameter, mm	21.25 ± 3.78	20.86 ± 3.55	0.702*
Initial NRS pain score	7.25 ± 1.26	7.55 ± 1.45	0.428*

*Student *t* Test, **Mann Whitney *U* Test, NRS: numerical rating scale.

**Table 2:** Comparison of the findings in women with gestational pure hydronephrosis followed by conservative treatment or by the insertion of double J stent.

	Double J inserted (*n* = 24)	Conservative treatment (*n* = 29)	*P* value
NRS pain score one week after the first admission	4.17 ± 1.37	4.31 ± 1.36	0.705*
Urinary tract infection after the first week, *n* (%)	9 (37)	10 (34)	0.824*
Urinary culture positiveness after the first week, *n* (%)	5 (21)	2 (6)	0.140**
Pyelonephritis after the first week, *n* (%)	5 (21)	4 (14)	0.501**
Renal function deterioration after the first week, *n* (%)	5 (21)	3 (10)	0.293**
Gestational week at birth	38	38	0.515*
The largest renal calyceal diameter at postpartum sixth week, mm	4.33 ± 3.87	4.83 ± 5.15	0.993**
Postpartum severe hydronephrosis (>15 mm) at postpartum sixth week, *n* (%)	1 (4)	2 (7)	0.672**
NRS pain score at postpartum sixth week	2.46 ± 1.44	2.03 ± 1.24	0.255*

*Student *t* Test, **Mann Whitney *U* Test, NRS: numerical rating scale.
